# Interferon (IFNγ) induces cell cycle arrest in RCC cell lines

**DOI:** 10.1186/2051-1426-1-S1-P125

**Published:** 2013-11-07

**Authors:** David J Tate, John R Patterson, Beatriz Finkel-Jimenez, Arnold H Zea

**Affiliations:** 1 Stanley S. Scott Cancer Center, LSUHSC-NO, New Orleans, LA, USA; 2 Microbiology Immunology and Parasitology, LSUHSC-NO, New Orleans, LA, USA

## 

We have recently demonstrated that the anti-proliferative effect of interferon (IFNγ) in RCC is due to an increased production of nitric oxide (NO) which in turn inhibits the induction of arginase (ARG2). The growth of cultured murine RCC cell lines was inhibited in 85% in IFNγ treated when compared to un-treated cells. In contrast only 33% of these cells were inhibited by IFNγ. These results indicate that even though IFNγ show a more potent anti-proliferative effect, still there is a certain degree of treatment- resistance (15%) that is higher when cells are treated with IFNγ. This study was undertaken to study the mechanisms by which IFNγ inhibits RCC growth and to provide new insights in understanding how this cytokine alone or in combination with other therapies could benefit RCC clinical outcome. Cell survival, cell cycle arrest-apoptosis and cell signaling targets were identified by MTT assay, flow cytometry and immunoblotting respectively in RCC cell lines CL-2 (low-ARG/low-iNOS), CL-19 (high-ARG/low iNOS) and Renca (med-ARG/zero-iNOS). The results showed that 1-100 U/ml IFNγ suppressed proliferation of both CL-2 and CL-19 in a dose- and time-dependent manner. IFNγ inhibited the growth of CL 2 and CL 19 tumor cells with IC50-values of 10.2 ± 1.5 U/ml and 13.5 ± 1.0 U/ml, respectively. There was no inhibition of growth in the Renca cell line. The results on cell cycle progression have shown that the anti-proliferative effect of IFNγ is associated with a G2/M cell cycle arrest of CL-2 and CL-19 cells. The event of cell cycle arrest was associated with a marked decline in protein levels of G2/M regulatory proteins cyclin B1, cyclin A and cyclin-dependent kinase 1 (Cdk1/cdc2), whereas an up-regulation in CDC25c was observed. Elimination of IFNγ resulted in increased tumor growth that was contained with the sustained addiction of the cytokine. No apoptosis was observed. Our study demonstrated that IFNγ suppress the growth of murine RCC cells by inducing cell-cycle arrest at G2/GM through the down-regulation of cyclin B1 and cyclin C. These findings suggest that IFNγ acts as an effective anti-proliferative agent by modulating cell growth regulators in renal carcinoma cells and can be used as a election treatment to inhibit tumor proliferation in patients scheduled for surgery.

